# The Perioperatively Altered Neutrophil-to-Lymphocyte Ratio Associates with Impaired DNA Damage Response in Liver Transplantation Recipients with Hepatocellular Carcinoma

**DOI:** 10.3390/diagnostics11020209

**Published:** 2021-01-30

**Authors:** Kuang-Den Chen, Chien-Ning Hsu, Yi-Ju Wu, Chi-Hsiang Chu, Kuang-Tzu Huang, Ming-Chao Tsai, King-Wah Chiu, Ben-Chung Cheng, Chien-Hua Chiu, Chao-Long Chen, Chih-Che Lin

**Affiliations:** 1 Liver Transplantation Center, Department of Surgery, Kaohsiung Chang Gung Memorial Hospital, Chang Gung University College of Medicine, Kaohsiung 83301, Taiwan; ding8570@gmail.com (K.-D.C.); wuyiju0904@gmail.com (Y.-J.W.); kuangtzu.huang@gmail.com (K.-T.H.); clchen@cgmh.org.tw (C.-L.C.); 2 Institute for Translational Research in Biomedicine, Kaohsiung Chang Gung Memorial Hospital and Chang Gung University College of Medicine, Kaohsiung 83301, Taiwan; 3 Department of Pharmacy, Kaohsiung Chang Gung Memorial Hospital and Chang Gung University College of Medicine, Kaohsiung 83301, Taiwan; cnhsu@cgmh.org.tw; 4 Department of Statistics, National Cheng-Kung University, Tainan 70101, Taiwan; loveweib@gmail.com; 5 Division of Hepato-Gastroenterology, Department of Internal Medicine, Kaohsiung Chang Gung Memorial Hospital and Chang Gung University College of Medicine, Kaohsiung 83301, Taiwan; tony0779@yahoo.com.tw (M.-C.T.); kwchiu@cgmh.org.tw (K.-W.C.); 6 Division of Nephrology, Department of Internal Medicine, Kaohsiung Chang Gung Memorial Hospital and Chang Gung University College of Medicine, Kaohsiung 83301, Taiwan; benzmcl@gmail.com (B.-C.C.); e22209@cgmh.org.tw (C.-H.C.)

**Keywords:** hepatocellular carcinoma, liver transplantation, neutrophil-to-lymphocyte ratio, DNA damage response, alcohol dehydrogenase, lymphopenia

## Abstract

Increasing evidence has suggested that elevated systemic inflammation with a high neutrophil-lymphocyte ratio (NLR) is associated with poor prognosis after liver transplantation (LT). The ongoing molecular events involved in poor survival remain unclear. This retrospective study evaluated LT recipients whose data was collected at Kaohsiung Chang Gung Memorial Hospital between 2005 and 2014. Clinical records of 347 patients with hepatocellular carcinoma from seven days before LT to 30 days after LT illustrated that longitudinal values of lymphocytes, RBC, and hemoglobin were persistently low in patients with peritransplant high NLR (PTH-NLR, pre-LT ≥ 4 and post-LT ≥ 5), which indicated a significantly worse survival rate in association with increased RDW-CV and pancytopenia when compared to other patients (*p* = 0.008). We further found that PTH-NLR patients had decreased DNA damage response (DDR) genes and detoxifying enzymes of ADH and ALDH families, and increased mitochondrial stress response genes in their liver tissues. Reduced lineage markers of liver progenitor cells were also observed in PTH-NLR patients signifying the presence of unresolved impairments after LT. Our results demonstrate the association between hematopoietic deficiencies and lack of protection against DDR with PTH-NLR in LDLT recipients with HCC and may imply abnormal hematological and organismal defects in those patients.

## 1. Introduction

The global prevalence of end-stage liver disease (ESLD) has been rising over the past several decades, with hepatocellular carcinoma (HCC) being the most common cause of death in ESLD and the third leading cause of cancer mortality worldwide. HCC is considered a heterogeneous disease whose major etiological causative factor in Asia is chronic infection with hepatitis viruses. Liver transplantation (LT) is considered the best treatment available for HCC patients suffering liver cirrhosis around the world. HCC patients who meet the agreed upon criteria, particularly Milan and UCSF criteria, were able to achieve a 5-year survival rate of above 70% [1]. LT provides the radical advantage of removing not only liver tumors but the residual organ underlying the risk of future malignancy [2]. Due to organ shortages, the practice of living donor liver transplantation (LDLT) is a treatment of choice for patients with developing tumors and has been safely extended to the salvage settings meeting the criteria during recent decades [3]. Although the survival rate after LDLT has drastically improved, patient death caused by poor patient status without HCC recurrence remains a significant problem [4]. In contrast, some HCC recipients that do not meet the criteria may have favorable outcomes [5]. Therefore, the optimal selection criteria and the potential molecular events that affect prognosis of HCC recipients after LDLT should be investigated.

Systemic inflammation is a critical part of tumor progression [6]. It is correlated with the poor prognosis of certain cancers; meanwhile, circulating immune-inflammatory cells such as platelets, neutrophils, and lymphocytes, have been found to play significant roles in those malignancies [7]. The presence of systemic inflammatory response results in changes in these cells, particularly the neutrophil-to-lymphocyte ratio (NLR) and the elevation of C-reactive protein (CRP) levels have been reported to serve as post-LT prognostic predictors for HCC patients [8]. Considerable research efforts have been devoted to demonstrating that NLR and the ratios of other combinations of inflammatory cells, including platelet-to-lymphocyte ratio (PLR) and lymphocyte-to-monocyte ratio (LMR), are useful in predicting the survival of various solid tumors, including HCC [9,10]. However, these indicators may not accurately reflect the inflammatory status and immune responses in patients due to a lack of understanding of the molecular events involved in changes of these immune cells. Furthermore, systemic inflammation triggered by oxidative stress is the cause of macromolecular damage and genome instability, which facilitates cancer progression and development.

The complicated DNA damage response (DDR) machineries that respond to repair highly toxic double strand breaks (DSBs) and inter-strand crosslinks (ICLs) are resolved by nucleotide excision repair (NER) and a more complex repair reaction involving the Fanconi anemia protein complexes [11]. The accumulation of genotoxic damage was thought to be elicited by a large variety of endogenous and exogenous stimuli and associated with impaired DDR and repair system [12]. Accumulated DNA lesions have been found to be accompanied by loss of cellular functionality, in the end, with abnormalities in the self-renewal and differentiation of stem cells from hematopoietic and tissue-specific origins [13,14]. However, the link between an impaired DNA repair system and systemic inflammation is still unclear.

The aim of this study was to investigate the impact of peritransplant NLR (i.e., within 7 days pre- and 30 days post-LT) on the survival outcome of LT patients with HCC, and explore the associations between patient outcomes and DNA damage response genes derived from the liver biopsies and peripheral blood mononuclear cells (PBMC).

## 2. Materials and Methods

### 2.1. Patients, Study Design, and Clinical Samples

In this study, we analyzed a total of 347 HCC patients who received LDLT in the liver transplantation (LT) center at Kaohsiung Chang Gung Memorial Hospital, Taiwan from 1 January 2005 to 31 May 2014. Patients were included for long-term survival outcome if they were (1) aged at least 20 years old at the time of undergoing LT, (2) had a survival time longer than 3 months after LT, (3) had an HCC diagnosis confirmed by two experienced pathologists, and (4) were willing to provide their written informed consent for sample collection. The exclusion of short-term morality cases is essential to avoid greater variability of individual risks, and our focus was on the investigation of contributors in long-term survival outcomes of recipients under liver transplantation condition. The Kaplan–Meier survival analyses inclusive of the short-term mortality cases showed similar results with poor survival in the PTH-NLR group than the control group as shown in Supplementary Figure S1. All the liver graft biopsies were collected from recipients during LDLT. The liver tissues used for quantifying gene expression were histologically examined as non-cirrhotic and non-cancerous liver tissue.

### 2.2. Data Collection and Assessments

The study patients’ electronic medical records, including diagnosis, prescriptions, procedures, and laboratory results, were retrieved from the study setting from 1 January 2005 to 31 May 2014.

The primary study endpoint was overall survival (OS). Patients were followed from the date of LT (index date) until either death or 16 August 2018 (the last day of study follow-up). Baseline characteristics, comorbid conditions using Charlson comorbid index (CCI) [15], and the Model for End-stage Liver Disease (MELD) score [16] before liver transplantation were all recorded for analyses.

Blood tests, including complete blood count (CBC), C-reactive protein (CRP) for inflammation markers, serum creatinine (SCr), blood urea nitrogen (BUN) for renal function, albumin (indicating nutrition status), total bilirubin, aspartate aminotransferase (AST), alanine aminotransferase (ALT), lactate dehydrogenase (LDH), prothrombin time (PT) and its international normalize ratio (INR) as markers of liver function, and electrolytes, were routinely checked in our center, and the laboratory results were retrieved from the ISO-certified laboratory center in the study setting at: ≤7 days prior to the index date, and 30 days after the index date.

CBC consists of WBC (white blood cells), RBC (red blood cells), hemoglobin, hemotocrit (HCT), red cell distribution width-coefficient of variation (RDW-CV), platelets (PLT), neutrophils (Segment), and lymphocytes. Inflammation markers included the neutrophil to lymphocyte ratio (*NLR*), and lymphocyte counts. The cutoff level of lymphocytes for severe lymphopenia was classified as lymphocytes < 500 and ≥500/μL, which was determined based on clinical relevance [17]. The determination of the cutoff point for the NLR is described in the Section 2.4.

### 2.3. RNA Isolation and Quantitative RT-PCR

Clinical samples of quality-verified RNA extracted from liver tissues were prepared using the RNeasy kit from Qiagen (Valencia, CA, USA) in accordance with the manufacturer’s instructions. The total RNA was eluted in 100 µL nuclease-free water, and precipitated by mixing eluate with 3 M final concentration of ammonium acetate, followed by subsequent ethanol addition (4×) and then chilled to −20 °C overnight.

Furthermore, 1 μg high-quality total RNA and the High Capacity Reverse Transcriptase (Applied Biosystems; Grand Island, NY, USA) were used in first strand cDNA synthesis. The quantitative Real-Time PCR was performed by using the TaqMan Fast Advanced Master Mix with specific TaqMan Assay probes (Thermo Fisher Scientific; Waltham, MA, USA) on an ABI 7500 fast PCR system (Applied Biosystems, Foster City, CA, USA). Amplifications were performed with an initial 95 °C for 20 s, followed by 40 cycles of 95 °C for 3 s and 60 °C for 30 s. Relative quantification of mRNA expression was calculated against GAPDH using the ∆∆Ct method.

The mRNA expression of DDR genes, mitochondrial stress response genes, alcohol dehydrogenases (ADHs), aldehyde dehydrogenases (ALDHs), cell surface markers of adult-derived liver stem cells, and DDR activation markers was examined in preoperative liver samples (20 cases for each group). The TaqMan probe sets that we used in the quantitative RT-PCR are listed in Supplementary Table S1.

### 2.4. Statistical Analysis

Continuous variables were expressed as mean ± standard deviation (SD), and we examined the differences between two subgroups using the independent *t* test. Categorical variables were expressed as numbers (*n*) with percentage (%), while subgroup differences were evaluated using the Chi-square test.

To identify the impact of NLR and predictors of LT survival in patients with HCC, we recorded and evaluated the potential prognostic factors between one week before and one month after LT. Pre- and post-LT risk factors of the study cohort were analyzed, and a time-dependent receiver operating characteristic (ROC) analysis was used to determine the appropriate cutoff values for outcome prediction. Because cut-off points of 4 for NLR ≤ 7 days prior to the date of LT (pre-NLR) and 5 for NLR ≤ 30 days after the date of LT were identified as a potential survival predictor, patients were classified into two groups according to pre-NLR and post-NLR levels (pre-NLR ≥ 4 & post-NLR ≥ 5, persistently high NLR group) versus others as control. We employed the Kaplan–Meier approach with log-rank tests employed for time to death event analysis. Cox proportional hazard model (adjusted hazard ratio (HR) with 95% confidence interval (CI)) was performed to justify the associations between pre-NLR/post-NLR and overall survival by other potential independent variables. A univariate variable with a *p*-value ≤ 0.1 of significance was included in the analysis of multivariate model. Two-tailed tests with *p* < 0.05 were considered statistically significant. For mRNA expression analysis using quantitative RT-PCR, statistical significance was determined using the Mann–Whitney *U* test against the control group. Processing and analyses of data were conducted using SAS Enterprise Guide v 5.1 (SAS, Cary, NC, USA) and SPSS 15.0 software (SPSS Inc.; Chicago, IL, USA).

## 3. Results

### 3.1. Patient Characteristics and NLR Predictors

The median follow-up was 6.9 years (ranging from 0.3 to 13.5 years) in 347 HCC patients who received LDLT. The overall 1-, 3-, and 5-year patient survival rates were 95.7%, 92.5% and 89.5%, respectively. The median age of study patients when receiving LDLT was 55 years (ranging from 26 to 69). Table 1 presents laboratory results within 7 days before LT and 30 days after LT.

**Figure 1:**
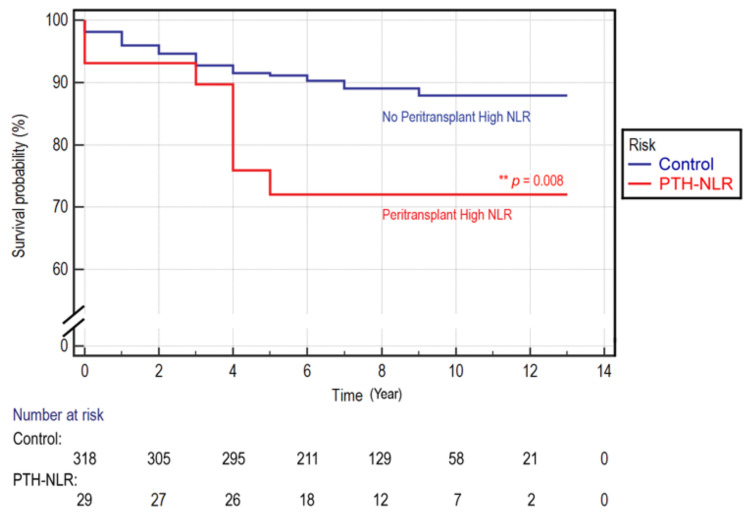
Kaplan–Meier overall survival curve in LT patients of HCC. A survival comparison for LT patients with or without peritransplant high NLR values. The mean survival for the patients with peritransplant high NLR (PTH-NLR) versus the non-persistent controls was 11.84 ± 0.19 versus 10.21 ± 0.85. The 1-, 3- and 5-year overall survival rates of control versus PTH-NLR group were 95.9%, 92.8%, and 91.1% versus 93.1%, 89.7%, and 72.1%, respectively (*p* = 0.008).

We found that the cutoff value of NLR determined by receiver operating characteristic (ROC) analysis was higher in post-LT (cutoff = 5) than that in pre-LT (cutoff = 4).The univariate analysis (crude HR) results showed that lymphocyte counts, SCr, and NLR values in the pre-LT period, and serum sodium, CRP, red cell distribution-coefficient of variation (RDW-CV), NLR, PLR, and LMR in the post-LT period were significantly associated with OS (Table 1). In the Cox regression analysis, a peritransplant high NLR (PTH-NLR, pre-LT NLR ≥ 4 and post-LT NLR ≥ 5) was significantly associated with an increased risk of all-cause mortality (adjusted HR, 4.565, 95%CI, 1.58–13.2; *p* = 0.005), whereas only a serum sodium level lower than 138.5 mmol/L was identified as an independent variable with significant association (*p* = 0.004). Kaplan–Meier survival analyses demonstrated that patients in the PTH-NLR group were more likely to have a poor survival rate than the control group with non-persistently high NLR values: 3-year OS (89.7% vs. 92.8%) and 5-year OS (72.1% vs. 91.1%), respectively (*p* = 0.008, Figure 1).Over the study period, the death event was 27.6% in the PTH-NLR group and 10.7% in the control group.

We explored the associations between high NLR levels (pre-LT NLR and post-LT NLR) and patient’s baseline clinical characteristics. Table 2 shows that the proportion of patients with higher CRP (≥5), and higher RDW-CV (≥15) were significantly greater in patients of higher values in pre-LT and post-LT NLR (both *p* < 0.05). Similarly, patients with a higher MELD score and more comorbid conditions in liver disorders were positively associated with patients of higher values in pre- and post-NLR (both *p* < 0.05). In contrast, the proportion of patients with lower hemoglobin (Male < 13.5, Female < 12 g/dL), and lower albumin (<3.5 mg/dL) occurred significantly greater in patients of higher values in pre- and post-NLR (both *p* < 0.05). The proportion of patients with higher levels of liver AST and ALT, as well as the presence of decompensated cirrhosis were significantly associated with patients of higher values in pre-NLR (*p* < 0.05). The results indicate that the liver function of patients with even higher values in post-NLR could be improved, however their elevated inflammation according to the persistently higher RDW-CV, CRP, and lower hemoglobin levels still remained at 1 month after LDLT.

### 3.2. Peripheral Blood Abnormality in Patients with Persistently High NLR Levels

**Figure 2:**
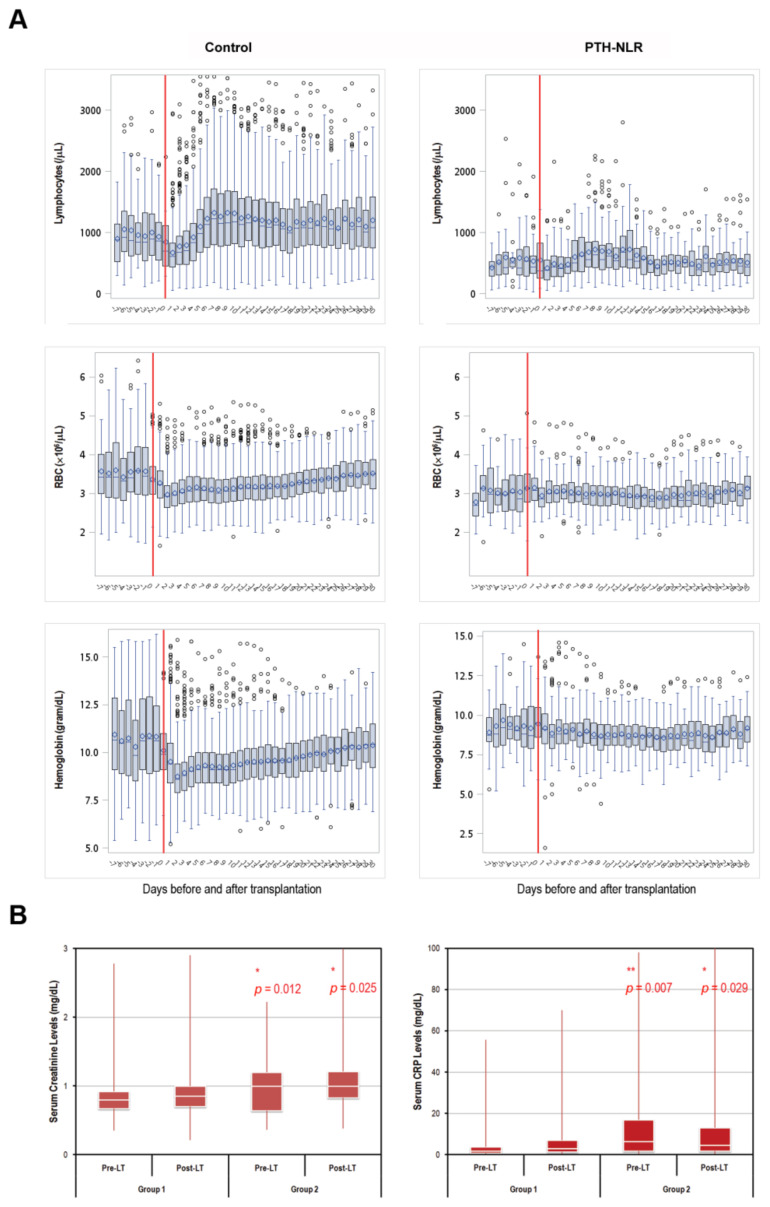
Trends of peritransplant lymphocytes, RBC, hemoglobin, creatinine, and CRP levels in LT patients of HCC with or without peritransplant high NLR. (**A**) The longitudinal lymphocyte and RBC counts as well as hemoglobin levels between 7 days pre-transplant and 30 days post-transplant were significantly lower in the PTH-NLR patients. Recovery of these values within one month after LT was remarkable in those patients without persistently high NLR (control group). (**B**) The Levels of both serum creatinine and CRP 7-day pre-transplant and 30-day post-transplant were significantly increased in PTH-NLR patients compared to those values in control patients. Significance is established at a *p*-value of 0.05 by using Mann–Whitney *U* test (*, ** for *p* < 0.005, *** for *p* < 0.001).

We further analyzed laboratory results between patients with persistently high NLR levels (PTH-NLR group) and those in the control group. As shown in Figure 2A, longitudinal values of lymphocyte counts, RBC, and hemoglobin from 7 days before LT to 30 days after LT were continuously low in the peritransplant high NLR group compared with the patients in the control group. On the other hand, sustained higher serum creatinine and CRP levels were also observed in the PTH-NLR group compared with control (Figure 2B).

**Figure 3:**
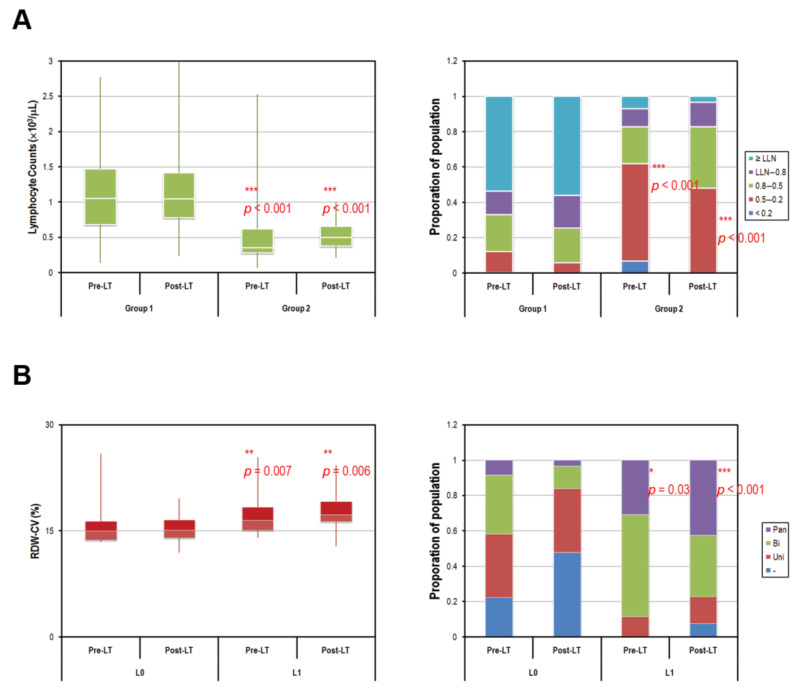
Comparison of pre- and post-transplant lymphocyte counts of HCC patients according to peritransplant NLR values. (**A**) Persistent low lymphocyte counts with a drastically high proportion of severe lymphopenia (<500/μL) were observed in peritransplant high NLR patients of HCC. The average 7-day pre- and 30-day post-transplant lymphocyte counts were 1091.5/μL and 1137.6/μL in control patients versus 536.6/μL and 540.5/μL in patients of PTH-NLR group, respectively (*p* < 0.0001, left panel). The proportions of pre- and post-transplant patients with severe lymphopenia were both less than 12% in control, however, those values were more than 48% (62.1% and 48.3%, respectively) in PTH-NLR group (red box, right panel). (**B**) Patients with persistently severe lymphopenia (PSL) represented increased RDW-CV values (left panel) and significantly larger proportions of pancytopenia (purple box, right panel) compared to the remaining patients (Control). LLN: lower limit of normal (1 × 10^3^/μL); Pan: pancytopenia; Bi: bicytopenia; Uni: unicytopenia. Significance is established at a *p*-value of 0.05 by using Mann–Whitney *U* test (*, ** for *p* < 0.005, *** for *p* < 0.001).

Figure 3A shows that the pre-LT and post-LT lymphocyte counts were significantly lower in PTH-NLR group compared with those in the control group. The proportions of pre-LT and post-LT lymphocytes <10^3^/μL were 46.4% and 44%, respectively, in control, whereas the proportions were significantly higher (93.1% and 96.6%) in PTH-NLR group (*p* < 0.001, Figure 3A, left panel). The proportions of pre-LT and post-LT lymphocytes <500/μL were both less than 12% in the control group; however they were more than 48% (62.1% and 48.3%, respectively) in the peritransplant high NLR group (*p* < 0.001, Figure 3A, right panel). Furthermore, HCC patients with peritransplant severe lymphopenia (<500/μL) represented increased RDW-CV (pre-LT: 16.5% vs. 15.1%, *p* = 0.004 and post-LT: 18.1% vs. 16.7%, *p* = 0.003, Figure 3B, left panel). Moreover, significantly higher proportions of pancytopenia were also observed among patients with continuously severe lymphopenia compared to the remaining controls (pre-LT: 30.8% vs. 13.4%, *p* =0.030 and post-LT: 42.3% vs. 4.1%, *p* < 0.001, Figure 3B, right panel).

### 3.3. Impairment in the Expression of Genes Involved in DNA Damage Repair Pathways in Patients with Persistently High NLR Levels

**Figure 4:**
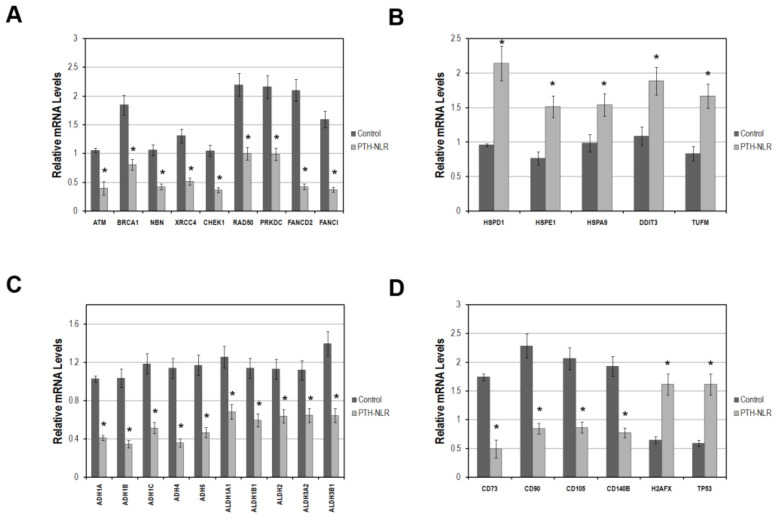
The mRNA levels of genes related to DNA damage response, mitochondrial stress response, metabolic detoxification, lineage markers on liver-derived stromal cells, and DDR activation markers in LDLT recipients with HCC. (**A**) DNA damage response genes were significantly reduced in recipients’ liver tissues with PTH-NLR. (**B**) This drastic increase of the mitochondrial stress genes was also observed in liver tissues of PTH-NLR recipients compared to those in control subjects. (**C**) The detoxifying genes of alcohol and aldehyde dehydrogenase families were significantly reduced in recipients’ liver tissues of PTH-NLR group compared to those in control. (**D**) The mean levels of four major lineage markers on liver stem cells were significantly decreased, whereas the DDR activation markers TP53 and H2AFX increased in the patients’ liver tissues of PTH-NLR group compared with those control recipients. Data represents 15 independent cases in each group using GAPDH as a reference gene for internal control. Significance is established at a *p*-value of 0.05 (*) by the Mann–Whitney *u* test.

To determine whether DNA repair genes may be involved in the largely increased cases of lymphopenia after LT, the mRNA expression of known DDR genes was examined in preoperative liver tissues of recipients before LT (20 cases for each group). As shown in Figure 4A, the DDR genes involved in both DSBs and ICLs repair pathways, which were ATM, BRCA1, NBN, XRCC4, CHEK1, RAD50, PRKDC, FANCD2, and FANCI were significantly lower in the liver tissues of LT patients in PTH-NLR group compared with those in the control group. The impaired ICLs repair has been linked to augmented mitochondrial stress that depends on increased oxidative stress and dysregulated mitochondrial activity. Interestingly, we also found that these five mitochondrial stress genes (HSPD1, HSPE1, HSPA9, DDIT3, and TUFM) in recipients’ liver tissue were drastically higher in the PTH-NLR group compared with those in the control group (Figure 4B). Moreover, the expression levels of alcohol and aldehyde dehydrogenase gene families were further examined in recipients’ liver tissues. As shown in Figure 4C, the mRNA levels of major alcohol dehydrogenases (ADH1A, ADH1B, ADH1C, ADH4, and ADH5) and aldehyde dehydrogenases (ALDH1A1, ALDH1B1, ALDH2, ALDH3A2, and ALDH3B1) were significantly lower in the liver tissues of recipients in PTH-NLR group. Our results indicated that impairment of DNA damage and repair responses could exist in those patients with peritransplant high NLR values.

Next, we investigated the cell surface marker genes expressed by liver-derived mesenchymal stem cells in the biopsies of recipients’ liver tissues. As shown in Figure 4D, the expression of lineage markers on liver progenitor cells CD73, CD90, CD105, and CD140B was significantly lower while the activation markers of DDR (TP53 and H2AFX) were increased in the liver tissues of recipients in PTH-NLR group. These results were consistent with the tendency of largely increased proportion of peritransplant lymphopenia subjects in the PTH-NLR group that might indicate a systemic impairment in DDR and the related detoxifying machinery.

## 4. Discussion

Liver transplantation is the ideal treatment for HCC patients who meet the standard criteria because it provides the best prognosis and eliminates not only the tumor but also the underlying liver disease. With the introduction of the Milan criteria in 1996, the prognosis in overall survival and tumor recurrence rate has greatly improved and is equivalent to that of non-HCC patients [18]. However, many HCC patients are not eligible for LT due to the strict candidate assessment. Therefore, many centers have been trying to expand the selection criteria to accept more HCC patients eligible for LT [19,20]. Growing evidence has shown that the systemic immunological inflammation index NLR, which is based on peripheral neutrophil and lymphocyte counts, is associated with poor outcomes in patients with several types of malignancies. Pretreatment NLR has been found to predict overall survival after LT in patients with HCC and correlated with functional indicators of the liver, such as the MELD score, Child Pugh score, and total bilirubin rather than the parameters of the Milan criteria, and the usefulness of NLR as a biomarker in HCC patients beyond the Milan criteria has also been suggested [21]. The relationship between post-LT NLR and graft size in adult-to-adult living donor liver transplantation (AA-LDLT) has also been reported, and acute post-LT NLR is a significant risk factor of 1-year mortality after AA-LDLT [22]. These studies demonstrated a relationship among higher NLR immediately after LT, low platelet count, and a poorer prognosis after LT. However, the mechanisms underlying the changes in NLR before and after LT remain unknown. Therefore, exhaustive and accurate investigation into NLR and the potentially involved molecular events are necessary for LT patients with HCC.

The present study reviewed our long-term experience of LDLT in HCC patients and accessed the association between peritransplant NLR, patients’ outcomes and, for the first time, potential impaired DNA damage events. We found that both high pre-LT and post-LT NLR values were associated with a vast decline in basal lymphocytes, but there was no significant increase in neutrophil counts in LDLT recipients with HCC. The clinical relevance of NLR represents a combination of factors related to both inflammation and immunity in LT recipients. Inflammation is a critical process thought to be associated with the up-regulation of cytokines and inflammatory mediators, which promotes the survival of tumor cells and elicits a hypoxic microenvironment for angiogenesis. Neutrophils are a major source of oncostatin M (OSM) and vascular endothelial growth factor (VEGF), and facilitate metastasis by promoting the adhesion and migration of tumor cells on liver sinusoidal cells [23]. However, the neutrophil counts were not associated with elevated NLR, nor were they correlated with poor prognosis in HCC patients after LT. Such results may indicate that the neutrophil counts did not exceed a certain threshold that would cause pathological outcomes even in HCC patients who exhibited high NLR values before and after LT. Furthermore, a high proportion of patients with a drastic drop in lymphocyte count was observed in the persistently high NLR group before and after LT (sees Figure 3A). Although the proportion of severe lymphopenia (<500/μL) was 62.1% at day-7 before LT and almost half (48.3%) at one month after LT in PTH-NLR recipients, only the pre-LT lymphocyte count was observed in this study as a prognostic factor in liver transplant recipients as described in Nagai’s previous study [17]. In our study, peritransplant lymphopenia plays a predominant role in elevated NLR and has been demonstrated to be prognostic in mortality after LDLT.

The liver is where the biosynthesis of proteins functionally important to the lipid metabolic and hematopoietic systems occurs. Synthetic dysfunction in the liver can have adverse effects on the maintenance of blood cell production. Therefore, hematological abnormalities such as cytopenia in any or all of the cell types are commonly observed in patients with liver disorders [24]. Although the underlying mechanisms are still largely unknown, the definitive management of hematological anomalies is critical for patients who undergo liver transplantation. In our study, lymphocyte counts were negatively associated with high NLR levels both before and after LT, and the proportion of pancytopenia in HCC patients with persistently severe lymphopenia after LT was significantly increased while in those without persistent lymphopenia, it was significantly reduced (See Table 2 and Figure 3B, right panel). This result indicates that about 7.5% of HCC patients in this cohort who might not recover from severe lymphopenia after LT have worse outcomes with regard to developing pancytopenia. Based on our observed association of hematological abnormalities with persistently high NLR, as well as the post-LT value of RDW-CV, other prognostic factors in HCC recipients, namely the changes of DDR genes and detoxifying enzymes, which are responsible for ensuring genomic integrity and genotoxic metabolization, respectively, may play an important role in these defects as suggested by Walter and his colleagues [25]. ICLs prevent the strand separation required for DNA replication and transcription by covalently linking the double helix together and are highly toxic [26]. In this study, we found that a number of DDR genes that have been implicated in the incision events of ICL repair were significantly lower in the liver tissues of PTH-NLR recipients, and the decrease of those DDR genes was also observed in peripheral blood cells of PTH-NLR recipients even after LT (data from an unpublished manuscript in preparation). A concomitant decrease in a series of ADHs and ALDHs in liver biopsies of PTH-NLR recipients was also observed. These enzymes are known to decrease oxidative stress particularly caused by oxidation-produced aldehydes [27]. Aldehydes are generated by various biochemical pathways essential to the living process, such as the metabolism of alcohols, amino acids, and drugs, inducing the production of hundreds of highly-reactive aldehyde species [28]. The accumulation of endogenous aldehydes is cytotoxic, which can lead to abnormal development, bone marrow failure, and cancer malignancies. These adverse effects can be scavenged by detoxifying enzymes, which have been the main focus of recent research in patients of a rare hereditary Fanconi anemia and animals with impaired expression of the DDR gene FANCD2 and the detoxifying enzymes ALDH2 and ADH5 [29,30]. Such research has demonstrated that endogenous formaldehyde is scavenged by ADH5, while the accumulation of formaldehyde-crosslinked adducts in DNA is characterized in ADH5 knockout mice [30]. While mice with combined deficiency in ADH5 and the corresponding repair gene FANCD2 would result in blood pancytopenia, the reduction of bone marrow cellularity and the acceleration of HSC attrition cause failure to sustain hematopoiesis. Furthermore, the accumulation of endogenous formaldehyde in ADH5 and FANCD2 double knockout mice causes damage beyond normal hematopoiesis. This previous study also demonstrated that a lack of protection against aldehydes leads to dysfunction in the liver and kidney. Mice with double knockout in ALDH2 and FANCD2 have also been found to spontaneously develop severe HSC attrition and bone marrow failure [29]. One study recently proved that the DNA crosslinking repair pathway is essential for loss of blood homeostasis in response to aldehydes [31]. Detoxifying enzymes like ALDH2 provide primary protection against aldehydes, whereas the Fanconi anemia pathway is an essential mechanism for counteracting those damages. Our study demonstrates that the peritransplant measurement of NLR predicts the overall survival of HCC recipients after LT, and the association of hematopoietic deficiencies lacking protection against DNA damages might also imply endogenous defects in those LT recipients. The limitation of this study is the lack of hematopoietic stem cell harvesting from LDLT recipients to examine the potential defects in DDR genes. Further investigation is required to determine the nature of the defects leading to hematopoietic deficiencies in patients with peritransplant high NLR values. The cutoff values of NLR prior- and post-liver transplantation, and the composite NLR (PTH-NLR) were based on the study cohort in a single center, future research is needed to further validate these study results. Although the PTH-NLR showed a statistically significant association with mortality, potential unmeasured confounders could have an impact on the analysis.

Our present study is the first to identify the association of peritransplant high NLR with concomitantly impaired gene expression involved in DDR and detoxification of aldehydes in recipients with HCC. The aberrant expression of DDR and detoxifying genes may also be responsible for the poor survival in recipients with persistently high NLR values. Therefore, identifying the sources and molecular details of DNA damage response is necessary for advancing the clinical care of liver transplant patients for HCC treatment, and may have important implications for cancer predisposition and the development of precision therapies.

## Figures and Tables

**Table 1:** Laboratory results and associations with all-cause mortality in the study cohort.

Variables ^ǂ^	Observations (%)	Univariate Model			Multivariate Model *		
		HR	95% CI	*p*-Value	HR	95% CI	*p*-Value
**Male**	290 (83.6)	1.377	0.29–1.85	0.502	0.745	0.28–1.98	0.555
Age at LT < 57 yr	214 (61.7)	1.381	0.70–2.71	0.348	0.983	0.94–1.03	0.477
*Pre*-*operative 7 days:*							
Lymphocytes < 500/μL	70 (20.2)	2.176	1.12–4.23	0.013			0.232
Sodium < 140	99 (28.5)	1.455	0.77–2.74	0.247			
CRP ≥ 1.8	190 (54.8)	1.049	0.55–1.99	0.884			
Total Bilirubin ≥ 2.65	55 (15.9)	1.842	0.90–3.77	0.094			
Direct Bilirubin ≥ 1.25	43 (12.4)	1.778	0.82–3.86	0.146			
Albumin < 3.5 g/dL	231 (66.6)	1.816	0.84–3.95	0.132			
SCr ≥ 1.5 mg/dL	15 (4.4)	3.250	1.27–8.27	0.014			0.181
RDW-CV ≥ 15%	171 (49.3)	1.774	0.93–3.40	0.084			
Related inflammatory scores:							
NLR ≥ 4	53 (15.3)	2.129	1.06–4.26	0.033			0.408
PLR ≥ 136	38 (11)	2.203	0.93–4.39	0.075			0.160
LMR < 5.7	57 (16.4)	1.587	0.31–1.30	0.215			
*Post*-*operative 30 days*							
Lymphocytes < 1000/μL	213 (42.7)	1.654	0.84–3.25	0.1437			
Sodium < 138.5	90 (25.9)	2.956	1.59–5.51	<0.001	2.515	1.347–4.695	0.004
CRP ≥ 5.9	113 (32.6)	2.340	1.27–4.32	0.006			0.309
Total Bilirubin ≥ 1.2	49 (14.1)	1.696	0.81–3.56	0.162			
Direct Bilirubin ≥ 0.3	163 (47)	1.430	0.77–2.67	0.261			
Albumin ≥ 4.5 g/dL	8 (2.3)	3.215	0.99–10.4	0.052			0.201
SCr ≥ 0.8 mg/dL	234 (67.4)	1.507	0.72–3.17	0.279			
RDW-CV % ≥ 15%	255 (73.5)	3.059	1.09–8.59	0.034			0.100
Related inflammatory scores:							
NLR ≥ 5	82 (23.6)	3.057	1.50–6.25	0.002			0.339
PLR ≥ 180	111 (32)	1.942	1.05–3.60	0.034			0.255
LMR < 2	63 (18.2)	2.268	1.17–4.39	0.015			0.197
Persistent high NLR(pre-LT NLR ≥ 4–post-LT NLR ≥ 5)	29 (8.4)	3.193	1.04–9.85	0.003	4.565	1.58–13.2	0.005

^ǂ^ The cutoff point of laboratory results for each variable was determined by receiver operating characteristic (ROC) analysis; * Factors in univariate model with significant at the 0.1 level were included in the Cox regression model.

**Table 2:** Patient characteristics by pre-NLR and post-NLR.

Characteristics	Pre-NLR Level		*p*-Value	Post-NLR Level		*p*-Value
	<4 (*n* = 283)	≥4 (*n* = 64)		<5 (*n* = 285)	≥5 (*n* = 62)	
**Gender**						
Female	45 (15.9%)	12 (18.75%)	0.5785	46 (16.14%)	11 (17.74%)	0.7577
Male	238 (84.1%)	52 (81.25%)		239 (83.86%)	51 (82.26%)	
**Complete blood count blood test ≤ 7 days before the index date**						
Lymphocytes						
<500	38 (13.43%)	35 (54.69%)	<0.0001	48 (16.84%)	25 (40.32%)	<0.0001
≥500	245 (86.57%)	29 (45.31%)		237 (83.16%)	37 (59.68%)	.
Segment, count						
<1000	47 (16.61%)	6 (9.38%)	0.1463	45 (15.79%)	8 (12.9%)	0.567
≥1000	236 (83.39%)	58 (90.63%)		240 (84.21%)	54 (87.1%)	
RBC, count						
Male < 4.3/Female < 3.9	188 (66.43%)	42 (65.63%)	0.902	184 (64.56%)	46 (74.19%)	0.146
Male ≥ 4.3/Female ≥ 3.9	95 (33.57%)	22 (34.38%)		101 (35.44%)	16 (25.81%)	
RDW-CV, count						
<15	143 (50.53%)	13 (20.31%)	<0.0001	138 (48.42%)	18 (29.03%)	0.0054
≥15	140 (49.47%)	51 (79.69%)		147 (51.58%)	44 (70.97%)	
HCT						
Male < 41/Female < 36	224 (79.15%)	50 (78.13%)	0.8556	225 (78.95%)	49 (79.03%)	0.9881
Male ≥ 41/Female ≥ 36	59 (20.85%)	14 (21.88%)		60 (21.05%)	13 (20.97%)	
MCV						
<80	30 (10.6%)	7 (10.94%)	0.9372	32 (11.23%)	5 (8.06%)	0.4645
≥80	253 (89.4%)	57 (89.06%)		253 (88.77%)	57 (91.94%)	
MCH						
<26	34 (12.01%)	8 (12.5%)	0.9143	35 (12.28%)	7 (11.29%)	0.8285
≥26	249 (87.99%)	56 (87.5%)		250 (87.72%)	55 (88.71%)	
MCHC						
<31	13 (4.59%)	6 (9.38%)	0.1289	13 (4.56%)	6 (9.68%)	0.1086
≥31	270 (95.41%)	58 (90.63%)		272 (95.44%)	56 (90.32%)	
Hemoglobin, g/dL						
Male < 13.5/Female < 12	193 (68.2%)	57 (89.06%)	0.0008	195 (68.42%)	55 (88.71%)	0.0013
Male ≥ 13.5/Female ≥ 12	90 (31.8%)	7 (10.94%)		90 (31.58%)	7 (11.29%)	
Platelet (×1000/μL)						
<40	63 (22.26%)	18 (28.13%)	0.3166	65 (22.81%)	16 (25.81%)	0.6129
≥40	220 (77.74%)	46 (71.88%)		220 (77.19%)	46 (74.19%)	
**Liver function (≤7 days) before the index date**						
Total bilirubin (mg/dL)						
<1.4	133 (47%)	37 (57.81%)	0.118	136 (47.72%)	34 (54.84%)	0.3095
≥1.4	150 (53%)	27 (42.19%)		149 (52.28%)	28 (45.16%)	
Differential bilirubin (mg/dL)						
<0.4	99 (35.74%)	21 (32.81%)	0.6585	97 (34.77%)	23 (37.1%)	0.7283
≥0.4	178 (64.26%)	43 (67.19%)		182 (65.23%)	39 (62.9%)	
LDH						
Male < 225/Female < 214	181 (69.88%)	40 (65.57%)	0.5123	185 (71.15%)	36 (60%)	0.092
Male ≥ 225/Female ≥ 214	78 (30.12%)	21 (34.43%)		75 (28.85%)	24 (40%)	
AST						
<37	83 (29.33%)	18 (28.13%)	0.0055	82 (28.77%)	19 (30.65%)	0.5047
37−111	187 (66.08%)	36 (56.25%)		186 (65.26%)	37 (59.68%)	
≥111	13 (4.59%)	10 (15.63%)		17 (5.96%)	6 (9.68%)	
ALT						
<40	169 (59.72%)	37 (57.81%)	0.0299	168 (58.95%)	38 (61.29%)	0.7891
40~120	107 (37.81%)	21 (32.81%)		107 (37.54%)	21 (33.87%)	
≥120	7 (2.47%)	6 (9.38%)		10 (3.51%)	3 (4.84%)	
CRP						
<5	223 (81.39%)	28 (43.75%)	<0.0001	214 (77.54%)	37 (59.68%)	0.0037
≥5	51 (18.61%)	36 (56.25%)		62 (22.46%)	25 (40.32%)	
						
Albumin, mg/dL						
<3.5	140 (49.47%)	53 (82.81%)	<0.0001	148 (51.93%)	45 (72.58%)	0.003
≥3.5	143 (50.53%)	11 (17.19%)		137 (48.07%)	17 (27.42%)	
**Renal function (≤ 7 days) before the index date**						
eGFR, mL/min/1.73 m^2^			0.9752			0.4889
<60	27 (14.14%)	6 (13.95%)		29 (14.8%)	4 (10.53%)	
≥60	164 (85.86%)	37 (86.05%)		167 (85.2%)	34 (89.47%)	
**Electrolytes (≤ 7 days) before the index date**						
Sodium (Na), mg/dL						
<148	274 (97.16%)	62 (98.41%)	0.5737	277 (97.54%)	59 (96.72%)	0.7175
≥148	8 (2.84%)	1 (1.59%)		7 (2.46%)	2 (3.28%)	
Calcium (Ca), mg/dL						
<10	266 (99.63%)	63 (100%)	0.6266	269 (99.63%)	60 (100%)	0.6368
≥10	1 (0.37%)	0 (0%)		1 (0.37%)	0 (0%)	
Chloride (Cl), mg/dL						
<112	219 (79.35%)	51 (80.95%)	0.7753	227 (81.65%)	43 (70.49%)	0.0599
≥112	57 (20.65%)	12 (19.05%)		51 (18.35%)	18 (29.51%)	
Potassium (K), mg/dL						
<3.6	81 (28.83%)	17 (26.56%)	0.6508	79 (27.82%)	19 (31.15%)	0.6449
3.6~5	197 (70.11%)	47 (73.44%)		202 (71.13%)	42 (68.85%)	
≥5	3 (1.07%)	0 (0%)		3 (1.06%)	0 (0%)	
**Comorbid conditions (≤1 year before the index date)**						
CCI score						
0	4 (1.41%)	5 (7.81%)	0.0082	5 (1.75%)	4 (6.45%)	0.0007
1–2	21 (7.42%)	7 (10.94%)		17 (5.96%)	11 (17.74%)	
≥3	258 (91.17%)	52 (81.25%)		263 (92.28%)	47 (75.81%)	
Congestive heart failure	1 (0.35%)	0 (0%)	0.6339	0 (0%)	1 (1.61%)	0.0318
Cerebral vascular disease	1 (0.35%)	0 (0%)	0.6339	1 (0.35%)	0 (0%)	0.6404
Chronic pulmonary diseases	5 (1.77%)	2 (3.13%)	0.4852	5 (1.75%)	2 (3.23%)	0.4552
Ulcer	35 (12.37%)	4 (6.25%)	0.1617	30 (10.53%)	9 (14.52%)	0.3674
Liver disease	271 (95.76%)	57 (89.06%)	0.0334	273 (95.79%)	55 (88.71%)	0.0264
Diabetes without complications	43 (15.19%)	8 (12.5%)	0.5825	43 (15.09%)	8 (12.9%)	0.6598
Diabetes with complications	2 (0.71%)	1 (1.56%)	0.5042	3 (1.05%)	0 (0%)	0.4172
Chronic kidney disease	7 (2.47%)	3 (4.69%)	0.339	6 (2.11%)	4 (6.45%)	0.0638
Severe liver diseases	38 (13.43%)	10 (15.63%)	0.6456	40 (14.04%)	8 (12.9%)	0.815
Decompensated cirrhosis	120 (42.4%)	45 (70.31%)	<0.0001	131 (45.96%)	34 (54.84%)	0.2048
**Basal characteristics**						
*n*	283	64		285	62	
Age at the index date						
mean	53.83 (7.59)	55.03 (5.83)	0.1638.	54.05 (7.32)	54.06 (7.32)	0.988
median	55 (49–59)	54 (51–59.5)		55 (50–59)	55 (50–60)	
Lymphocytes						
mean	1070 (500)	579 (400)	<0.0001	1033 (510)	732 (491)	<0.0001
median	1010 (688–1440)	454 (307–754)		983 (608–1426)	629 (345–963)	
Segment, count						
mean	1893 (951)	2981 (2509)	0.001	2056 (1209)	2269 (2202)	0.828
median	1806 (1135–2426)	2449 (1325–3829)		1836 (1172–2601)	1665 (1224–2690)	
SCr						
mean	0.85 (0.3)	0.84 (0.37)	0.9322	0.86 (0.31)	0.81 (0.34)	0.2671
median	0.8 (0.65–0.95)	0.8 (0.61–0.97)		0.8 (0.67–0.95)	0.78 (0.54–0.94)	
MELDScore						
mean	10.04 (2.99)	13.17 (5.29)	<0.0001	10.33 (3.3)	11.92 (5.11)	0.0215
median	9.28 (7.7–11.84)	11.57 (9.71–15.8)		9.46 (7.76–12.04)	10.34 (8.45–13.93)	

Pre-NLR level was based on the ratio of neutrophil-lymphocyte obtained ≤ 7 days before the index date (the date of liver transplantation); and post-NLR level was obtained ≤30 days after the index date; The independent *t* tests were performed for continuous data and chi-squared tests were performed for categorical data. Cutoff point was based on the normal range in clinical implications.

## Data Availability

Data available from authors.

## Supplementary Materials

The following are available online at https://www.mdpi.com/2075-4418/11/2/209/s1, Supplementary Table S1: List of probes and primers. The Applied Biosystems TaqMan assays consisting optimized target-specific primers and probes that were used for real-time PCR in Figure 4 are listed. (DOCX, 18 kb). Supplementary Figure S1: Kaplan–Meier overall survival analysis inclusive of the short-term mortality cases in LT patients of HCC. A survival comparison for LT patients with or without peritransplant high NLR values. The survival curve inclusive of the mortality cases who died within 3 months after LDLT showed similar results with poor survival in the PTH-NLR group than the control group (*p* = 0.001).

## Abbreviations

NLR, neutrophil-to-lymphocyte ratio; PTH-NLR, peritransplant high NLR; DDR, DNA damage response; HCC, hepatocellular carcinoma; LT, liver transplantation; RDW-CV, red cell distribution width-cofficience of variance; ADH, alcohol dehydrogenase; ALDH, aldehyde dehydrogenase; CRP, C-reactive protein; PLR, platelet-to-lymphocyte ratio; LMR, lymphocyte-to-monocyte ratio; ROS, reactive oxygen species; DSBs, double strand breaks; ICLs, interstrand crosslinks; OS, overall survival; CCI, Charlson comorbid index; MELD, model for end-stage liver disease; WBC, white blood cells; RBC, red blood cells; SCr, serum creatinine; BUN, blood urea nitrogen; AST, aspartate aminotransferase; ALT, alanine aminotransferase; LDH, lactate dehydrogenase; PT, prothrombin time; INR, international normalize ratio; HCT, hemotocrit; MCV, Mean corpuscular volume; MCH, mean corpuscular hemoglobin; MCHC, mean corpuscular hemoglobin concentration.
